# Outcomes of surgical, medication and combination therapies for cherubism: a systematic review and qualitative analysis

**DOI:** 10.3389/froh.2026.1740561

**Published:** 2026-04-29

**Authors:** Vera Julia, Lilies Dwi Sulistyani, Diwiya Aryyaguna, Revini Nuita, Elizabeth Shinta Maharani, Jovian Purnomo, Dwi Ariawan, Muhammad Adhitya Latief, Yudy Ardilla Utomo, Mohammad Farid Ratman, Norifumi Nakamura

**Affiliations:** Oral and Maxillofacial Surgery, Department of Oral and Maxillofacial Surgery, Faculty of Dentistry, University of Indonesia, Jakarta, Indonesia

**Keywords:** cherubism, combined modality therapy, giant cell lesion of the jaw, pharmacologic approach, *SH3BP2* mutation, surgical management

## Abstract

**Background:**

Cherubism is a rare genetic fibro-osseous disorder characterized by symmetrical expansion of the maxilla and mandible. The lesions usually progress during childhood, stabilize around puberty, and regress in adulthood. Treatment strategies depend on lesion progression, tissue involvement, and patient concerns. This systematic review aimed to summarize treatment approaches, including surgery, medication, and combined modalities, and to provide an overview of clinical, radiographic, microscopic, and molecular findings. Evidence-based treatment decision-making in cherubism remains challenging, as no prior systematic review has comprehensively evaluated and compared the outcomes of surgical, pharmacologic, and combined approaches.

**Methods:**

This review followed PRISMA guidelines. PubMed, ScienceDirect, Cochrane, and SpringerLink databases were searched for studies on cherubism management. Eligible studies included cohort studies, retrospective analyses, case series, and case reports. Late-onset and reactivation cases were excluded. Eighteen studies published between 2015 and 2025 were included after evaluation for relevance, risk of bias, and outcome reliability.

**Results:**

A total of 18 studies comprising 36 patients with cherubism were analyzed. The age ranged from early childhood (around 4 years) to young adulthood (up to 21 years). Genetic testing for *SH3BP2* mutations was reported in 5 studies, while a positive familial history was documented in 8 studies. Twelve patients were treated surgically using contouring, curettage, or resection. Seventeen patients received pharmacological therapy alone, including calcitonin, denosumab, or immunomodulating drugs. Seven patients underwent combined surgical and medical treatment. Reported outcomes varied across modalities.

**Conclusions:**

Cherubism management should be individualized based on disease grade and progression. Lower grades respond to conservative or pharmacologic therapy, whereas higher grades often require combined surgical and medical approaches using agents such as denosumab, imatinib, or tacrolimus. Advances in molecular insight and 3D-guided techniques support a trend toward personalized, grade-based treatment. Multicenter longitudinal studies remain essential to validate current strategies and establish standardized management guidelines for cherubism.

**Systematic Review Registration:**

https://www.crd.york.ac.uk/PROSPERO/view/CRD42024623376, PROSPERO CRD42024623376.

## 1 Introduction

Cherubism is a rare hereditary bone disorder characterized by symmetrical expansion of the jaws, in which giant cell lesions progressively replace normal bone tissue (1, 2). The disease, inherited in an autosomal dominant pattern, was first described in 1933 as a familial multilocular jaw condition and later termed cherubism because of the rounded, cherubic facial appearance reminiscent of Renaissance art (3, 4). Cherubism is categorized as a giant cell lesion caused by a missense mutation in the *SH3BP2* gene, which encodes an adapter protein involved in lymphocyte activation, osteoclast differentiation, and bone remodelling through signaling pathways involving protein kinases and NFATc1 (nuclear factor of activated T cells 1) (5, 6).

The disease typically manifests in early childhood (around 2 years of age), progresses rapidly until approximately age seven, stabilizes during puberty, and often regresses spontaneously thereafter (6). Clinically, cherubism presents as bilateral enlargement of the mandible, although the maxilla may also be affected (7). The severity and aggressiveness of the lesions vary widely, influenced by the degree of gene mutation penetration and expression (3, 7). Severe phenotypes are usually observed in younger patients and may result in significant facial deformity, dental malocclusion, or functional impairment (8).

Several grading systems have been proposed to classify the clinical severity of cherubism. Among them, the Raposo-Amaral system is a refined modification of the earlier Motamedi classification, integrating both clinical and radiographic features (2, 8). However, Chrcanovic et al. reported that the Motamedi system remains particularly relevant for assessing agenesis and radiographic characteristics, although it still lacks correlation with molecular findings (2, 9). Importantly, the inconsistency between genotype and phenotype expression complicates the understanding of mutation effects and their functional relationship with disease manifestation (2, 10, 11).

Despite growing understanding of the genetic and molecular basis of cherubism, therapeutic management remains largely empirical. Previous studies predominantly focused on surgical correction and conservative observation, whereas recent investigations have explored pharmacologic interventions targeting osteoclast regulation, such as calcitonin, bisphosphonates, and TNF-α inhibitors (11–16). However, these approaches have produced inconsistent outcomes due to the rarity of the disease, limited sample sizes, and heterogeneity in grading and follow-up assessment (17, 18). Systematic analyzes integrating clinical, radiological, and molecular data remain scarce, and the literature lacks a unified framework to compare outcomes across different treatment modalities (19). This persistent fragmentation underscores the need for an updated synthesis of evidence to clarify which management strategies yield the most predictable results across cherubism grades (20).

This absence of a clear genotype–phenotype correlation, coupled with the lack of a universally accepted grading system and standardized treatment protocol, contributes to significant variability in disease management (21). While most cases regress spontaneously, surgical intervention is typically reserved for patients with severe functional compromise, psychosocial distress, or an increased risk of pathological fracture (22). Pharmacologic and immunomodulatory approaches have been explored, yet their efficacy remains uncertain (23). Previous observational studies have reported inconclusive outcomes for drug-based therapies due to heterogeneity in study design and unclear treatment endpoints (8, 14, 21, 24). Current literature, dominated by case reports, describes a range of surgical and nonsurgical interventions with generally favourable outcomes but without consensus on the most effective regimen (25–28).

Previous studies on cherubism have largely focused on isolated treatment modalities or anecdotal case-based outcomes, without integrating surgical, pharmacologic, and combination therapies within a unified analytical framework. Most available reports describe individual cases or small series emphasizing short-term responses, making it difficult to draw comparative conclusions across treatment types or disease severities. Furthermore, earlier reviews have not systematically correlated therapeutic outcomes with clinical grading systems such as the Raposo-Amaral classification, resulting in a fragmented understanding of how lesion grade influences therapeutic response. Consequently, the literature lacks an integrated, grade-based comparative synthesis that could guide evidence-informed decision-making and standardize management protocols for cherubism.

Given the diversity of grading systems and treatment strategies, comparative assessment across studies remains difficult. To address this gap, the present systematic review classifies published cherubism cases according to the most recent Raposo-Amaral grading framework, incorporating clinical, radiological, and molecular data where available. The objective is to synthesize and qualitatively analyze existing treatment modalities, outcomes, and follow-up findings to provide a comprehensive understanding of cherubism management and to support the development of more standardized therapeutic guidelines.

## 2 Methods

This review was designed to systematically identify, appraise, and synthesize published evidence on the outcomes of surgical, pharmacologic, and combination therapies for cherubism. The methodology followed the Preferred Reporting Items for Systematic Reviews and Meta-Analyses (PRISMA 2020) framework and adhered to rigorous standards to ensure reproducibility and transparency. Each step, from literature search to data extraction and bias assessment, was conducted according to predefined eligibility criteria and registration protocol, as outlined in the following subsections.

### 2.1 Study design and protocol registration

The protocol was prospectively registered in the International Prospective Register of Systematic Reviews (PROSPERO) under the registration number CRD42024623376. The review design was developed to integrate both qualitative synthesis and descriptive analysis of outcomes from published reports on cherubism management, including surgical, pharmacologic, and combination therapies.

### 2.2 PICO framework and research questions

The research questions were formulated according to the PICO (Population, Intervention, Comparison, and Outcome) framework to ensure systematic and transparent synthesis. This structured approach facilitated clear identification of the patient population, intervention types, and measurable outcomes relevant to cherubism management. The framework used in this review is summarized in Table 1.

**Table 1:** PICO framework for the present review.

Component	Definition		Description
Population	(P)	Patients diagnosed with cherubism	Individuals of any age or sex with confirmed cherubism based on clinical, radiological, or histopathologic features
Intervention	(I)	Therapeutic or management strategies	Any form of treatment, including pharmacologic therapy (calcitonin, denosumab, imatinib), surgical intervention, or combined approaches
Comparison	(C)	Alternative or no treatment	Studies comparing surgical vs. non-surgical therapy, pharmacologic vs. observation, or multimodal vs. single-modality management
Outcome	(O)	Clinical, radiological, or functional response	Primary outcomes: lesion regression, bone remodeling, recurrence rate. Secondary outcomes: aesthetic improvement, airway function, quality of life, and adverse events

Based on this framework, the central research questions of the present systematic review were:

- What are the reported therapeutic strategies and clinical outcomes in patients with cherubism over the past decade (2015–2025)?
- How do different treatment modalities—pharmacologic, surgical, or combined—compare in terms of functional recovery, lesion regression, and recurrence prevention?
- What evidence-based recommendations can be derived to guide grade-specific management of cherubism?

### 2.3 Eligibility criteria

Studies were selected according to predefined inclusion and exclusion criteria. The detailed criteria are summarized in Table 2.

**Table 2:** Inclusion and exclusion criteria for study selection.

Criteria Type	Description
Inclusion Criteria	Publications between 2015 and 2025 Human subjects with a confirmed diagnosis of cherubism (clinical, radiological, or histopathologic) Studies reporting therapeutic interventions (surgical, pharmacologic, or combined) with documented clinical outcomes Eligible study designs: case reports, case series, and retrospective cohort studies
Exclusion Criteria	Non-human or *in vitro* research Review articles, commentaries, or conference abstracts Studies lacking treatment or follow-up outcome data Duplicate or overlapping patient cohorts without new information

All studies meeting these inclusion criteria progressed to full-text review and data extraction. These inclusion and exclusion criteria were designed to ensure the consistency and comparability of clinical outcomes across studies. The publication period (2015–2025) was intentionally limited to capture contemporary therapeutic developments, including the emergence of targeted pharmacologic therapies and advances in surgical techniques and 3D-based planning, thereby improving the clinical relevance and comparability of included studies. In addition, earlier studies frequently lacked standardized reporting of clinical outcomes, detailed treatment protocols, and consistent grading systems, which limited their eligibility for systematic comparison (1).

### 2.4 Search strategy and information sources

A systematic electronic search was performed to ensure comprehensive literature coverage in PubMed, ScienceDirect, Cochrane Library, Scopus, MDPI, and SpringerLink databases. The Boolean search string for PubMed was as follows:

[“cherubism”[Title/Abstract] OR “*SH3BP2”*[Title/Abstract]] AND (“treatment” OR “management” OR “therapy” OR “surgery” OR “pharmacologic” OR “denosumab” OR “calcitonin” OR “imatinib” OR “bisphosphonate”)

The final search was completed on April 15, 2025. Manual searches of the reference lists of included articles and relevant reviews were also conducted to capture additional eligible studies. No publication type filters were applied other than language and year. Search results were exported to Microsoft Excel for screening and duplication.

### 2.5 Study selection process

Two reviewers (NN and LDS) independently screened titles and abstracts of all retrieved records. Studies deemed potentially relevant underwent full-text review. Disagreements were resolved through discussion with a third reviewer (VJ). The selection process ensured that all included studies met predefined eligibility criteria, with consistent application across screening stages to maintain methodological rigor and reproducibility.

### 2.6 Data extraction and data items

Data extraction was performed using a standardized Microsoft Excel form developed for this review. Extracted variables included:

- Study characteristics: author, year, design, country, and sample size.
- Patient demographics: age, age at onset, and family history.
- Diagnostic parameters: radiological findings, histopathologic results, and genetic testing (*SH3BP2* mutation status).
- Interventions: type of therapy (surgical, pharmacologic, or combined), drug regimen, surgical procedure, and duration.
- Outcomes: lesion regression, functional improvement, aesthetic results, recurrence, adverse events, and follow-up duration.

Each dataset was verified by two reviewers for accuracy and completeness. Any missing or ambiguous data were annotated as “not reported” (NR). Data were extracted into a standardized sheet including study design, patient demographics, lesion grade (Raposo-Amaral), intervention type, treatment details, adverse events, and follow-up duration. For transparency, aggregated characteristics of the included studies are summarized in Table 3.

**Table 3:** Summary of included studies reporting cherubism cases (2015–2025).

Author (Year)	Study Design	Patients (n)	Onset Age (years)	Grade (I–VI)	Age of intervention (years)	Intervention Type	Follow-up (months)	Main Outcome
Kadlub et al. (29)	Case report	1	2	VI	4	Tacrolimus	12	Partial regression
Lenouvel et al. (22)	Case report	1	5	I	11	Debulking in mandible	6	Complete regression and satisfactory aesthetic result
Friedrich et al. (30)	Case report	1	1	III	17,19	Excision and LeFort I Osteotomy	132	Uneventful healing
Kugushev et al. (31)	Case report	1	5	VI	8.5	Denosumab	6	Partial lesion regression
Son et al. (9)	Case report	1	3	II	7	Excision mandible	15	Complete regression
Ricalde et al, (25)	Case series	3	6	II	Patient 1: 6	Imatinib	24	Marked partial regression (75%)
				III	Patient 2: 5;8	Mandible Debulking; Imatinib	10	Moderate partial response (22%)
				IV	Patient 3: 2;4	Mandible Debulking; Imatinib	10	Marked partial regression (65%)
Droma et al. (32)	Case series	2	NR	III, V	Patient 1:9	Denosumab	9	Marked partial regression (Patient 1)
					Patient 2: 11			Partial regression (Patient 2)
Bradley et al. (24)	Case report	1	6	V	13	Anterior maxillectomy + Alendronic acid	24	Partial regression
Morice, et al. (12)	Case report	1	3	II	6	Surgical resection	NR	No Complication
Kawamura et al. (33)	Case report	1	4.5	IV	10	Denosumab	24	Partial regression and ossification of lesion
Stoor et al. (28)	Case series	2	NR	V	Patient 3: 2; 4	Anti-TNF	91.5	Partial regression
					Patient 4: 3;10	Anti-TNF + Mandible buccal plasty	93	
Zoe et al. (7)	Case report	1	NR	V	7	Calcitonin	108	Complete regression, no regrowth
Jarvinen et al. (17)	Case Report	1	8	II	18	Reduction plasty	120	Partial Regression (70%)
Schreuder et al. (34)	Cohort Study	9	4	I, III, V	Patient 1: 6	Calcitonin	24	Partial Regression
					Patient 2: 7			(Patient 1,2,3,4,5,6,8,9)
					Patient 3: 7			No Regression (Patient 7)
					Patient 4: 8			
					Patient 5: 9			
					Patient 6: 10			
					Patient 7: 11			
					Patient 8: 12			
					Patient 9: 13			
Butts et al. (23)	Case Report	1	7	VI	8	Calcitonin + Debulking + Contouring	72	Complete regression and satisfactory aesthetic result
Sonpal et al. (21)	Case Report	1	12	VI	20	Imatinib	6	Partial regression and stable lesion
Peckan et al. (26)	Retrospective Study	7	NR	III-VI	Patient 3: 7	Surgical	62	Partial Regression
					Patient 4: 11	Resection and Contouring		
					Patient 5: 10			
					Patient 6: 11			
					Patient 7: 4			
					Patient 9: 4			
				VI	Patient 8: 8	Surgical Resection, contouring, Denosumab	86	Partial Regression
Ghawsi, et al. (4)	Case Report	1	11	IV	1	Surgical Contouring	216	No Complication

NR, Not recorded; Anti-TNF, Anti-Tumor Necrosis Factor.

## 3 Results

### 3.1 Study selection

**Figure 1:**
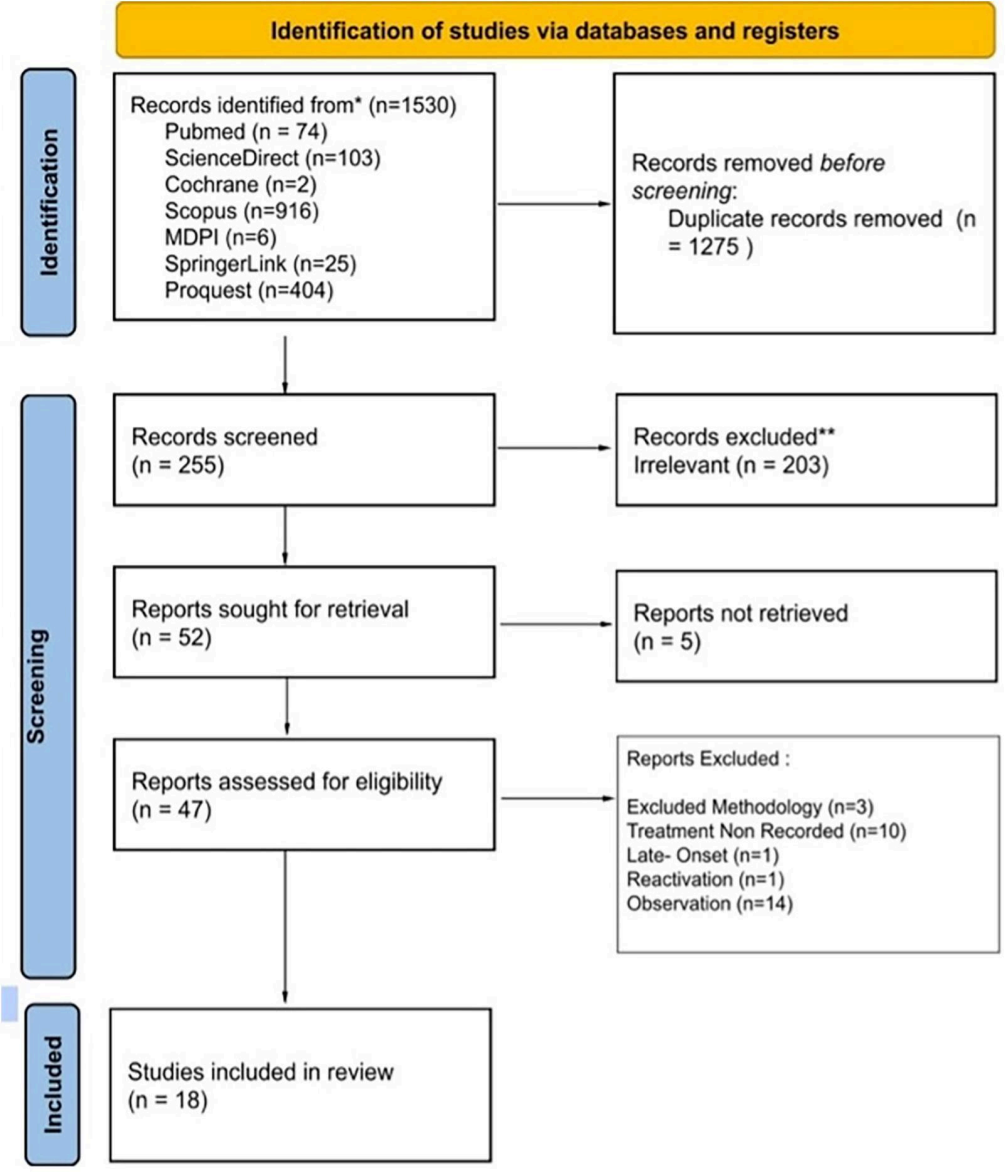
PRISMA flowchart.

The database search yielded 1,530 records across all sources. After removal of 1,275 duplicates, 255 records were screened based on title and abstract, of which 203 were excluded due to irrelevance. A total of 52 reports were sought for retrieval; 5 could not be retrieved, leaving 47 full-text articles assessed for eligibility. Of these, studies were excluded due to inappropriate methodology (*n* = 3), absence of treatment outcome data (*n* = 10), late-onset cases (*n* = 1), reactivation cases (*n* = 1), and observation-only studies (*n* = 14). Eighteen studies met the inclusion criteria and were included in the qualitative synthesis. The study selection process is summarized in the PRISMA flow diagram (Figure 1).

### 3.2 Study characteristics

A total of 18 studies encompassing 36 patients were included in this review. The characteristics of the included studies are summarized in Table 3, including study design, number of patients, age range, Raposo-Amaral grade, intervention type, follow-up duration, and principal outcomes. Overall, the included studies comprised case reports and case series with variable patient demographics and treatment approaches, reflecting the heterogeneity of clinical management in cherubism.

As shown in Table 3, most patients underwent surgical procedures alone or in combination with pharmacologic therapy. Combined approaches, particularly those incorporating denosumab, were associated with the most favourable regression rates and minimal recurrence, supporting the potential role of multimodal management strategies in cherubism.

The included studies comprised 10 case reports, 7 case series, and 1 retrospective study, published between 2015 and 2025. Most patients were children or adolescents (age range 4–25 years), reflecting the early onset of cherubism. Mandibular involvement was the most frequently reported lesion site, followed by combined mandibular–maxillary presentations in higher-grade cases.

Treatment approaches varied across studies, with the majority employing surgical management (curettage, contouring, or resection), while several utilized pharmacologic interventions such as calcitonin, denosumab, or imatinib. A small number of studies adopted observation-only strategies for mild (Grade I–II) lesions that demonstrated spontaneous regression.

Follow-up durations varied widely across studies, ranging from months to several years, with most studies reporting at least partial lesion regression and improved facial contouring. Notably, combined surgical–pharmacologic interventions consistently yielded superior outcomes, achieving the highest rates of lesion regression and the lowest recurrence rates (Table 3).

### 3.3 Classification and genetic findings

Lesions were classified according to the Raposo-Amaral grading system (Table 4), ranging from Grade I (localized mandibular involvement) to Grade VI (extensive craniofacial lesions). Among the 36 patients, 4 were Grade I–II, 7 were Grade III–IV, and 25 were Grade V–VI, demonstrating a predominance of moderate to severe disease.

**Table 4:** Cherubism grading system according to raposo-amaral et al. (28).

**Grade**	**Definition**
I	Lesion of the mandible without signs of root resorption
II	Lesion involving the mandible and maxilla without signs of root resorption
III	Aggressive lesion of the mandible showing signs of root resorption
IV	Lesion involving the mandible and maxilla showing signs of root resorption
V	The rare, massively growing, aggressive, and extensively deforming juvenile case involving the maxilla and mandible
VI	The rare, massively growing, aggressive, and extensively deforming juvenile case involving the maxilla and mandible, and orbits

Genetic analysis for *SH3BP2* mutation was reported in 5 studies (27.8%). Among these, 11 patients tested positive for pathogenic variants. A positive family history of cherubism was recorded in 8 studies (44%), supporting the autosomal dominant inheritance pattern. Radiographically, multilocular “soap-bubble” lesions and mandibular expansion were the most common findings (90%).

### 3.4 Risk of bias assessment

Risk of bias was evaluated using the Joanna Briggs Institute (JBI) critical appraisal tools specific for each study design, including case reports, case series, and retrospective cohorts. The detailed results of individual assessments are presented in Table 5, which outline the methodological quality across the included studies. Overall, 14 studies (77.8%) were rated as having a low risk of bias, 3 (16.7%) as moderate, and 1 (5.5%) as high risk. The most frequent methodological limitations were small sample sizes, lack of blinding in outcome assessment, and incomplete follow-up reporting. Despite these inherent constraints, the collective evidence was considered sufficiently robust to support the descriptive synthesis and comparative qualitative analysis presented in subsequent sections.

**Table 5:** Quality assessment of included studies with JBI critical appraisal tool (35).

Article	Study Design	Q1	Q2	Q3	Q4	Q5	Q6	Q7	Q8	Q9	Q10	Q11	Quality
Kadlub et al. (29)	Case Report	Y	Y	Y	Y	Y	Y	Y	Y	-	-	-	G
Lenouvel et al. (22)	Case Report	Y	Y	Y	U	Y	Y	Y	Y	-	-	-	G
Friedrich et al. (30)	Case Report	Y	Y	Y	Y	Y	Y	Y	Y	-	-	-	G
Kugushev et al. (31)	Case Report	Y	Y	Y	U	Y	Y	Y	Y	-	-	-	G
Son et al. (9)	Case Report	Y	Y	Y	Y	Y	Y	Y	Y	-	-	-	G
Bradley et al. (24)	Case Report	Y	Y	Y	U	Y	Y	Y	Y	-	-	-	G
Kawamura et al. (33)	Case Report	Y	Y	Y	Y	Y	Y	Y	Y	-	-	-	G
Zoe et al. (7)	Case Report	Y	Y	Y	Y	Y	Y	Y	Y	-	-	-	G
Jarvinen et al. (17)	Case Report	Y	Y	Y	Y	Y	Y	Y	Y	-	-	-	G
Butts et al. (23)	Case Report	Y	Y	Y	Y	Y	Y	Y	Y	-	-	-	G
Sonpal et al. (21)	Case Report	Y	Y	Y	Y	Y	Y	Y	Y	-	-	-	G
Ghawsi et al. (4)	Case Report	Y	Y	Y	Y	Y	Y	Y	Y	-	-	-	G
Ricarde et al. (25)	Case Series	Y	Y	Y	Y	Y	Y	Y	Y	Y	Y	-	G
Droma et al. (32)	Case Series	Y	Y	Y	Y	Y	Y	Y	Y	Y	NA	-	G
Stoor et al. (28)	Case Series	Y	Y	Y	Y	Y	Y	Y	Y	Y	Y	-	G
Schreuder et al. (34)	Retrospective	Y	Y	Y	U	U	Y	Y	Y	Y	U	Y	G
Pekcan et al. (26)	Retrospective	Y	Y	Y	U	U	Y	Y	Y	Y	Y	Y	G

Y, Yes; N, No; U, Unclear; NA, Not Applicable. Quality assessment was performed using Joanna Briggs Institute (JBI) critical appraisal tools according to study design.

A consolidated summary of the JBI quality appraisal across all study designs illustrates that, although methodological rigor varied among individual studies, the majority met essential quality criteria related to patient selection, diagnostic confirmation, and outcome clarity. The aggregated results confirm that most evidence sources demonstrate acceptable internal validity and sufficient reporting transparency to justify inclusion in the overall qualitative synthesis. Consequently, the comparative findings presented in the following section were interpreted in light of these quality assessments to ensure analytical consistency and reliability.

### 3.5 Treatment modalities and outcomes

Treatment strategies were categorized into three groups: surgical, pharmacologic, and combined surgical–pharmacologic interventions as further summarized in the subsequent section. Surgical management (*n* = 12) included contouring, curettage, partial resection, and reconstruction. Most surgical cases demonstrated favourable clinical outcomes, including lesion regression and improvement in facial symmetry, although a small number of cases reported recurrence during follow-up. Pharmacologic therapy (*n* = 17) included calcitonin, denosumab, imatinib, and anti-TNF agents, with variable responses ranging from partial to complete regression; denosumab was associated with radiologic improvement but required monitoring for calcium-related adverse effects. Combined therapy (*n* = 7), typically involving surgery followed by pharmacologic adjuncts, showed generally consistent improvement in both functional and aesthetic outcomes. Across all modalities, no mortality was reported, and adverse events were generally mild and manageable. Treatment selection appeared to be influenced by disease severity, patient age, and functional involvement.

### 3.6 Treatment outcomes and summary of findings

Building upon the preceding quality assessments, the synthesis of treatment outcomes revealed considerable variability in therapeutic approaches and clinical responses among the reviewed studies. Management strategies for cherubism were broadly categorized into three main modalities: (A) non-surgical or medication-based, (B) surgical, and (C) combination treatments. Each approach reflects different clinical rationales depending on lesion severity, patient age, aesthetic considerations, and functional impairment. Table 6 summarizes these therapeutic modalities and corresponding outcomes, illustrating the spectrum of clinical responses ranging from partial lesion regression to complete resolution with long-term stability.

**Table 6:** Treatment matrix for cherubism cases across reviewed studies.

Treatment Category	Article	Location	Grade	Pain	Swelling	Orbital Involvement	Airway Condition	Medication/Dosage/Duration	Surgical Treatment	Outcome	Adverse Events	Follow-up
A. Non-surgical/Medication	Kadlub et al. (29)	Bilateral maxilla and mandible	VI	No	Yes	Orbital compression	Glossoptosis, sleep apnea	Tacrolimus oral 0.15 mg/kg/day, twice daily	-	Stable and partial lesion regression, improved breathing	No	1 year
	Kugushev et al. (31)	Bilateral maxilla and mandible	VI	No	Yes	Exobirtalism	No	Denosumab oral 120 mg on days 8 and 15, then every 4 weeks (5 months)	–	Increased bone density, partial regression of lesion	No	6 months
	Droma et al. (32)	Bilateral mandible	III	No	No	No	No	Denosumab intracutaneous 120 mg on days 1, 8, 15, 28, then every 28 days (6 months)	-	Marked partial regression of lesion, Increased density of lesion	No	9 months
	Droma et al. (32)	Bilateral mandible	V	No	Yes	No	No	Denosumab intracutaneous 120 mg on days 1, 8, 15, 28, then every 28 days (6 months)	–	Partial regression of lesion	No	9 months
	Kawamura et al. (33)	Bilateral maxilla and mandible	IV	No	Yes	No	No	Denosumab intracutaneous 120 mg on days 0, 7, 28 then every 28 days for 6 months	–	Partial regression and ossification of lesion	Mild hypo/hypercalcemia, reduced growth rate	22 years
	Zoe et al. (7)	Bilateral maxilla and mandible	V	No	Yes	No	No	Calcitonin intranasal 200 IU/day (6 months)	–	Complete resolution and regression of lesion, no regrowth	No	9 years
	Schreuder et al. (34)	Maxilla & mandible (8 patients), mandible (1 patient)	I, III, V	NA	NA	NA	NA	Calcitonin subcutaneous 100 IU/day for 2 years	–	Most patients showed partial regression with variable responses: poor (3, 8), limited (1, 4, 5, 9), and mild (2, 6). One patient (7) showed no regression and unclear benefit.	73% AEs: nausea, vomiting, site reaction, rare: hypocalcemia	2 years
	Ricalde et al. (25)	Bilateral mandible	V	Yes	Yes	No	No	Imatinib 200 mg once a day for 12 months	–	Marked partial regression of lesion (75%), resolved dysmorphology	No	2 years
	Stoor et al. (28)	Bilateral maxilla and mandible	VI	NR	Yes	NR	No	Anti-TNF for 2.5 years	–	Partial regression with near-normal facial appearance, attributed to normal midfacial growth and no further excessive chin growth.	No	7 years
B. Surgical	Lenouvel et al. (22)	Right unilateral mandible	I	No	Yes	No	No	–	Debulking in mandible	Satisfactory aesthetic result following complete regression	Lower right lip paresthesia	6 months
	Friedrich et al. (30)	Bilateral mandible	III	No	Yes	No	No	–	Excision and LeFort I osteotomy	Uneventful healing	No	11 years
	Son et al. (19)	Bilateral mandible	II	No	Yes	Bilateral lower lid scleral show	No	–	Excision of mandible, orbit, and maxilla (Caldwell-Luc)	Satisfactory aesthetic result following complete regression	No	15 months
	Morice et al. (12)	Bilateral maxilla and mandible	II	No	Yes	Exophthalmos with limited ocular mobility	Nasal obstruction, glossoptosis	–	Partial surgical resection	No complications	No	NR
	Jarvinen et al. (17)	Bilateral mandible	II	No	Yes	No	No	–	Reduction plasty using CAD-CAM	Partial regression (70%) with satisfactory aesthetics and mild asymmetry	Bilateral mental nerve hypoesthesia	10 years
	Pekcan et al. (26)	Maxilla and mandible (6 patients)	III–VI	Yes	Yes	Yes	Yes	–	Surgical resection and contouring (all 6 patients)	Partial regression and improved facial symmetry observed in all patients, resolution of proptosis and improved respiratory function noted in patient 9	Temporary anesthesia, mandibular abscess	5.1 years (mean)
	Ghawsi et al. (4)	Bilateral maxilla and mandible	IV	No	Yes	No	No	-	Surgical contouring	Satisfactory healing with normal IAN sensibility and no complication	No complication	18 years
C. Combination	Ricalde et al. (25)	Bilateral maxilla and mandible	VI	No	Yes	Upward gaze	OSA, epistaxis	Imatinib 300 mg once a day for 10 months	Debulking	Partial regression of lesion (22%), improved breathing	Nausea	10 months
	Ricalde et al. (25)	Bilateral mandible	V	No	Yes	No	No	Imatinib 300 mg once a day for 10 months	Debulking	Marked partial regression (65%), resolved dysmorphology	Nausea	10 months
	Bradley et al. (24)	Bilateral maxilla and mandible	V	No	Yes	No	Shortness of breath when lying flat	Alendronic acid 70 mg weekly for 6 months	Anterior maxillectomy	Partial regression	No	24 months
	Stoor et al. (28)	Bilateral maxilla and mandible	VI	NR	Yes	NR	No	Anti-TNF for 2.5 years	Buccal plasty of mandible	Partial regression and favorable outcomes, with no evidence of further excessive growth.	No	7 years
	Butts et al. (23)	Bilateral maxilla and mandible	VI	No	Yes	No	No	Calcitonin 200 IU intranasal daily for 12 months	Mandibular debulking & contouring with fat graft	Satisfactory aesthetics, no recurrence and complete regression of lesion	Mild hypocalcemia, mandibular abscess	6 years
	Sonpal et al. (21)	Bilateral maxilla and mandible	VI	No	Yes	Blurring of vision	No	Imatinib 200 mg once daily post-op	Malar bone shaving	Partial regression of lesion, improved symmetry, proptosis and breathing.	-	6 months
	Pekcan et al. (26)	Maxilla and mandible (1 patient)	VI	Yes	Yes	Yes	Yes	Denosumab	Surgical resection	Partial regression, improved facial symmetry, with additional resolution of proptosis and improvement in breathing	Rebound hypercalcemia	7.2 years

Treatment approaches were grouped into surgical, pharmacologic, and combined modalities (Table 6). Pharmacologic therapies, including denosumab, calcitonin, and imatinib, showed variable responses ranging from partial to complete regression. Surgical interventions were primarily performed to improve facial contour and function. Combined approaches generally demonstrated consistent clinical improvement, with low rates of adverse events.

**Figure 2:**
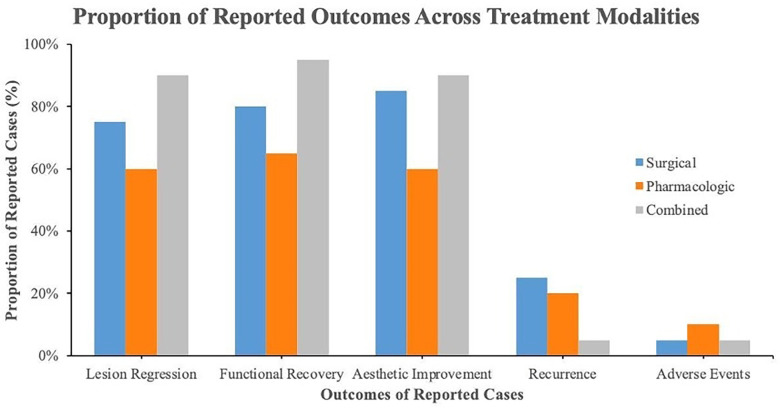
Comparative clinical outcomes across treatment modalities in cherubism.

Figure 2 presents a comparative overview of treatment outcomes across cherubism grades. Overall, combined surgical–pharmacologic therapy demonstrated the highest rate of sustained regression and functional restoration, particularly for Grade III–V lesions. Pharmacologic therapy alone was effective in early or mild cases (Grades I–II), while surgical treatment alone was reserved for cases with aesthetic or functional deformities. The collective findings indicate that cherubism management should be individualized according to disease grade and clinical progression, with integrated approaches yielding the most stable outcomes.

In summary, the collective findings highlight that therapeutic success in cherubism depends on a multidimensional approach integrating clinical assessment, lesion grading, and individualized treatment planning. The comparative evidence synthesized in Table 6 and Figure 2 demonstrates that combination strategies yield the most favourable long-term outcomes, while isolated pharmacologic or surgical interventions are effective only under specific clinical conditions. These observations underscore the evolving understanding of cherubism management and provide a foundation for the interpretative discussion in the following section.

This table compiles all reported treatment strategies for cherubism cases identified in the literature, grouped into three categories: (A) non-surgical/medication, (B) surgical, and (C) combination strategies. Each entry provides study reference, lesion location, grade, clinical symptoms, airway involvement, treatment regimen, outcome, adverse events, and follow-up duration.

## 4 Discussion

The objective of this study was to integrate data presented in case studies and literature (case reports, case series, and retrospective cohorts) to sharpen the overall understanding of cherubism, particularly in terms of treatment management, as well as to determine the results during the follow-up period. This review included 18 studies comprising 36 patients with cherubism, who were managed using surgical, pharmacologic, or combined treatment approaches between 2015 and 2025. Based on Raposo-Amaral et al., the management of cherubism depends on the degree of severity, especially for surgical modalities, and clinical data, radiographic findings, molecular analysis, and histology form the basis for classifying cherubism into the grading system developed by them (36). This grade-based approach provides an important foundation for determining the most appropriate therapeutic strategy for each patient (30, 36).

### 4.1 Overview of main findings

**Figure 3:**
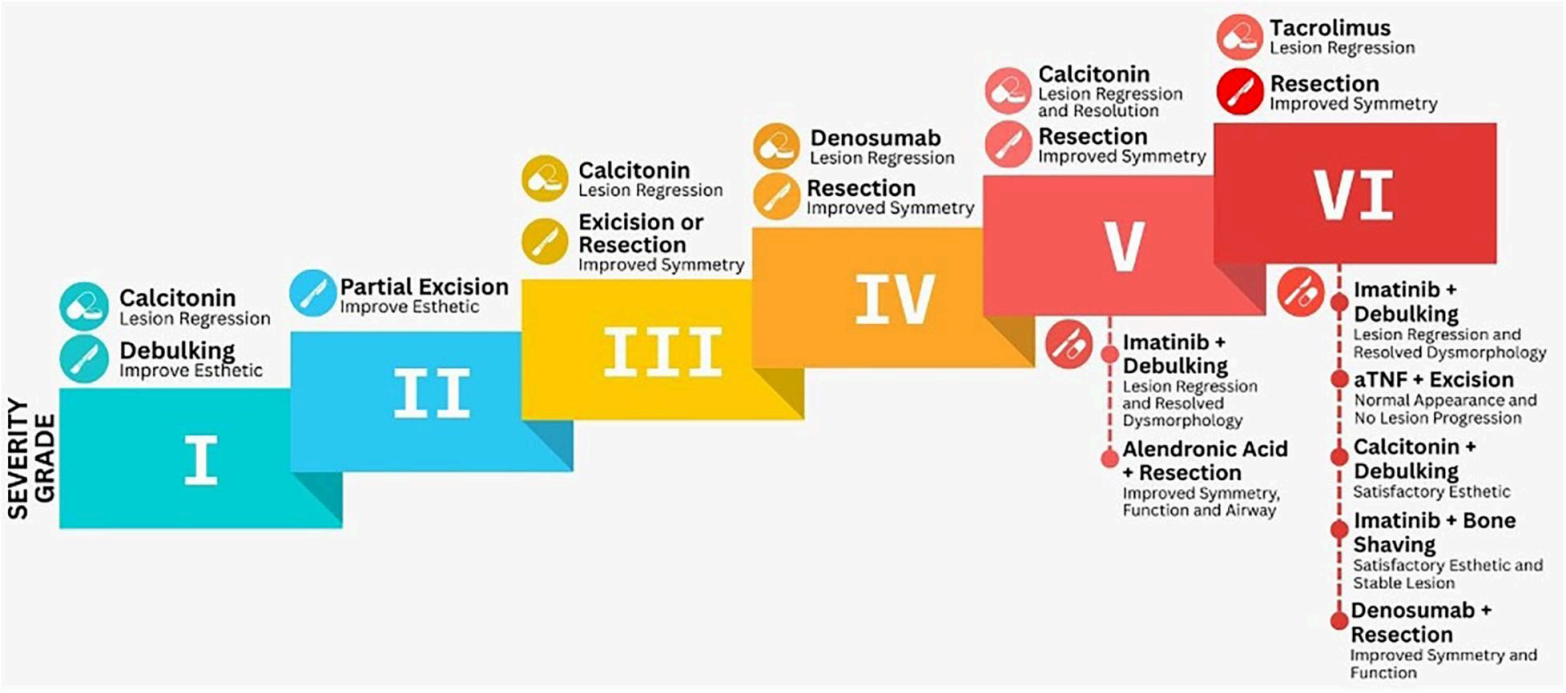
Overall synthesized systematic review findings.

The overall findings of the analysed literature are synthesized in Figure 3. Different treatment strategy adoption patterns are seen along the increasing disease severity. Surgical intervention was reported across all disease grades, with the extent of surgery increasing in proportion to disease severity. Pharmacological monotherapy appears in every grade except grade II, with the aim of controlling lesion development or halting its progression. In contrast, combination therapy is only used in advanced grades (V and VI), where pharmacological modulation is integrated with surgical intervention to optimize patients treatment outcomes (23–25, 28). Overall, these findings emphasize that personalized therapeutic planning based on disease's severity grading remains central to effective cherubism management, highlighting the importance of tailoring interventions to lesion behaviour and patient-specific factors (30). Building on this empirical pattern, the following section interprets the biological and mechanistic basis underlying the different therapeutic responses observed across modalities.

The synthesis of findings in this review demonstrates consistent patterns in therapeutic response across treatment modalities and disease grades. Compared with previous reviews, which primarily provided descriptive aggregations without explicit cross-modality comparison, the present analysis establishes a more structured, grade-based interpretation of outcomes. Chrcanovic et al. 2) systematically summarized clinical and molecular features of cherubism but did not address the comparative efficacy of different treatment modalities (1). Similarly, Cailleaux et al. (1) focused exclusively on pharmacologic interventions, concluding that evidence for denosumab and calcitonin remains inconclusive due to limited longitudinal data (2). In contrast, the current review integrates both surgical and pharmacologic approaches, highlighting how their combination achieves superior lesion regression and stability across moderate to severe disease grades.

Building upon these comparative insights, the following section (4.2) discusses the underlying biological and mechanistic basis for these observed therapeutic differences, explaining how pharmacologic modulation and surgical intervention interact synergistically to optimize clinical outcomes.

### 4.2 Interpretation and mechanistic insights

The predominance of favourable outcomes with combined therapy can be attributed to its dual mechanism of lesion control: surgical debulking of fibro-osseous tissue complemented by pharmacologic suppression of osteoclastic activity (23, 30, 37). Denosumab, a monoclonal antibody targeting RANKL, directly inhibits osteoclast differentiation and function, thereby attenuating giant-cell–mediated bone resorption within cherubic lesions (31–33). Calcitonin, in contrast, acts through the calcitonin-receptor pathway to stimulate osteoblast-driven remodelling and promote lesion stabilization (34). These pharmacologic actions synergize with surgical contouring, reducing recurrence risk and enhancing long-term structural normalization (37).

Nevertheless, rebound hypercalcemia and lesion reactivation reported after denosumab withdrawal underscore the importance of long-term monitoring and gradual dose tapering (34). Collectively, these observations support a biologically integrative framework of cherubism management, in which molecular modulation sustains and amplifies the benefits of surgical intervention (38).

**Figure 4:**
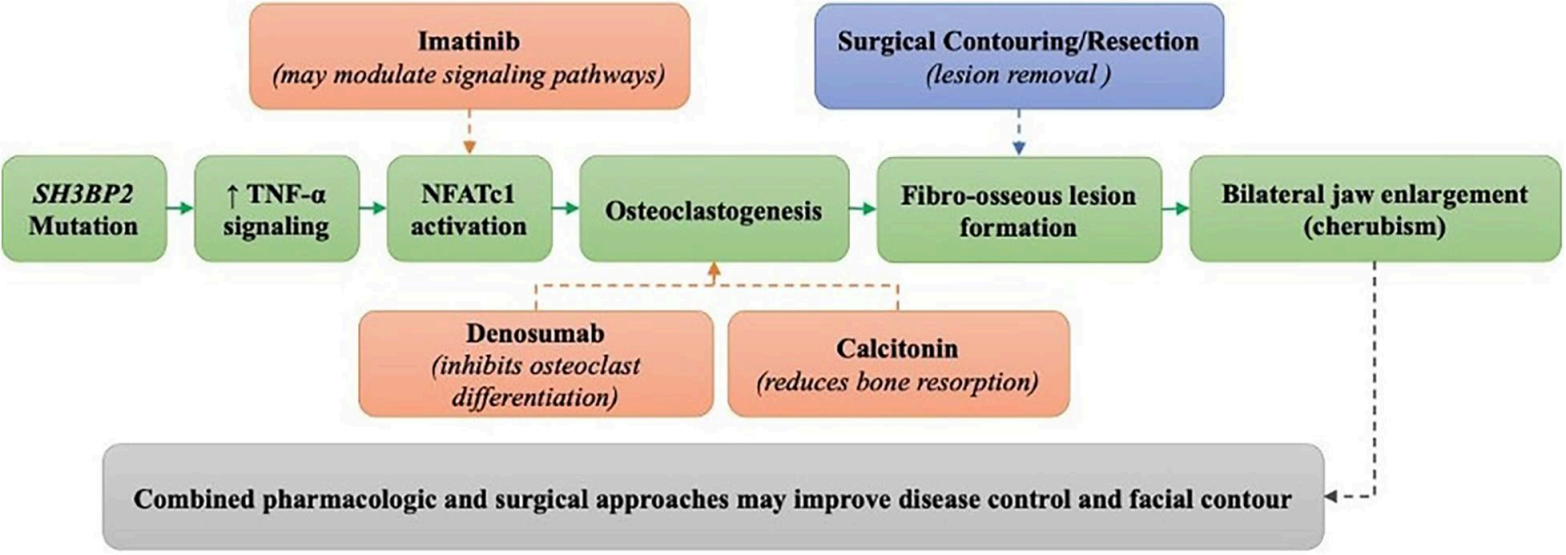
Pathophysiologic mechanisms of cherubism and therapeutic points of intervention. Mutations in the *SH3BP2* gene lead to hyperactivation of TNF-α and NFATc1 pathways, promoting osteoclastogenesis and fibro-osseous lesion formation. These molecular events result in bilateral jaw enlargement characteristic of cherubism. Pharmacologic therapies such as denosumab, calcitonin, and imatinib target osteoclastic activity, while surgery provides mechanical contouring. Combined approaches act at multiple levels, offering the more comprehensive disease control.

Figure 4 illustrates the proposed pathophysiologic model of cherubism, integrating genetic (*SH3BP2* mutations) and cellular (TNF-α, NFATc1) mechanisms driving osteoclast genesis and fibro-osseous proliferation. The diagram highlights how targeted pharmacologic therapy and surgical correction operate at complementary molecular and structural levels, thereby explaining their superior therapeutic synergy.

### 4.3 Clinical and research implications

The integrated synthesis of multidisciplinary evidence underscores the importance of precision-based, grade-specific therapeutic algorithms in cherubism management. For mild cases (Grades I–II), observation or pharmacologic monotherapy may be adequate, whereas moderate to severe forms (Grades III–V) benefit from multimodal regimens that combine surgery with antiresorptive or immunomodulatory agents. Clinically, early genetic confirmation of *SH3BP2* mutations, coupled with radiological grading, is critical for guiding treatment selection and anticipating disease trajectory (39). Advances such as three-dimensional (3D) surgical planning and computer-assisted contouring further enhance aesthetic precision while minimizing functional compromise, representing a valuable adjunct in contemporary practic (17).

**Figure 5:**
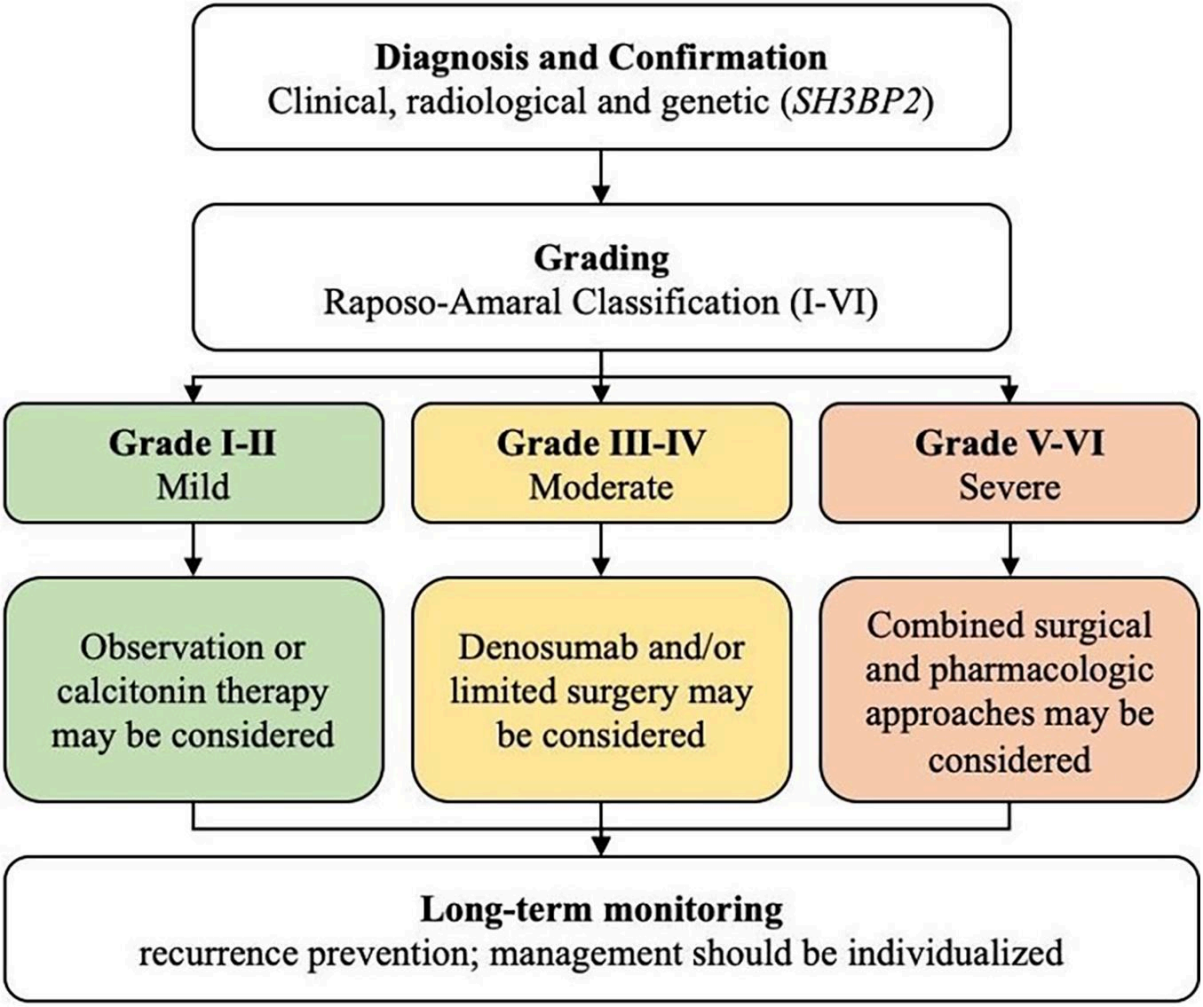
Grade-based treatment algorithm for cherubism management. The algorithm integrates diagnostic confirmation, disease grading, and therapeutic selection according to the Raposo-Amaral classification. Mild lesions (Grades I–II) may be managed conservatively or with calcitonin, while moderate forms (Grades III–IV) benefit from denosumab and limited surgery. Severe, disfiguring lesions (Grades V–VI) require combined surgical–pharmacologic approaches with long-term monitoring to prevent recurrence. This framework promotes evidence-based, individualized treatment planning.

From a research perspective, these findings highlight the need for standardized outcome metrics, including functional recovery, facial symmetry, and quality-of-life indices, to enable robust interstudy comparisons and future meta-analytic integration (40). The grade-based treatment framework derived from this review, illustrated in Figure 5, presents a stepwise decision pathway integrating disease severity, functional impact, and therapeutic modality. This algorithm translates current evidence into a practical guide for individualized cherubism management, aligning clinical decision-making with evidence-based principles and biological plausibility.

### 4.4 Limitations

Despite its comprehensive scope, this review has several limitations that should be considered when interpreting the findings. First, the available evidence is inherently constrained by the predominance of case reports and small case series, which limits statistical generalizability and increases susceptibility to publication bias. Second, the heterogeneity of reporting formats, particularly in lesion grading, treatment protocols, and follow-up duration, precluded quantitative meta-analysis and necessitated a descriptive synthesis approach. Third, incomplete or inconsistent documentation of radiological and functional outcomes across studies restricted direct comparison of therapeutic efficacy. Additionally, potential language and database bias may have excluded relevant non-English publications or unpublished data.

Nevertheless, these limitations are common in rare-disease literature and were mitigated through rigorous study selection, standardized bias appraisal, and structured qualitative synthesis. The identified gaps also inform future research priorities, emphasizing the need for multicenter collaboration, standardized reporting, and longitudinal outcome tracking, issues elaborated in the following section.

### 4.5 Future directions

Future research on cherubism should prioritize prospective, multicenter collaborations to expand sample sizes and capture the full clinical spectrum of the disease. Establishing standardized diagnostic and grading criteria, particularly integrating radiological, histopathologic, and genetic parameters, will be essential for ensuring comparability across studies. Longitudinal investigations incorporating functional, aesthetic, and psychosocial outcomes are needed to evaluate the long-term impact of different therapeutic modalities beyond short-term lesion regression.

Advances in molecular profiling may also enable the identification of predictive biomarkers for treatment response, facilitating personalized therapeutic planning and early intervention. The integration of artificial intelligence and 3D modeling into surgical simulation could further refine precision and outcome predictability. Finally, the development of international registries and open-access databases would strengthen evidence accumulation and accelerate translational progress in this rare disorder. Collectively, these future directions emphasize the transition from anecdotal case-based evidence toward data-driven, precision-oriented frameworks for cherubism management, bridging clinical experience with emerging biomedical technologies.

## 5 Conclusion

This systematic review consolidates contemporary evidence on the management of cherubism, highlighting the evolution from isolated surgical interventions toward multimodal, precision-based therapeutic strategies. The synthesis of 18 studies involving 36 patients demonstrates that combined surgical–pharmacologic approaches yield the most durable functional and aesthetic outcomes, particularly in moderate to severe cases. Pharmacologic therapy alone remains appropriate for early or indolent lesions, provided that treatment is guided by radiological and genetic assessment. Beyond clinical practice, these findings reinforce the importance of individualized, grade-specific treatment algorithms and standardized reporting frameworks to enhance comparability and cumulative knowledge. As research advances toward molecularly informed and technology-assisted care, cherubism management is poised to transition from anecdotal experience to evidence-driven, patient-centered precision therapy.

## Data Availability

The original contributions presented in the study are included in the article; further inquiries can be directed to the corresponding author.
